# The gene mutational discrepancies between primary and paired metastatic colorectal carcinoma detected by next-generation sequencing

**DOI:** 10.1007/s00432-018-2742-1

**Published:** 2018-08-31

**Authors:** Shuang-Mei Zou, Wei-Hua Li, Wen-Miao Wang, Wen-Bin Li, Su-Sheng Shi, Jian-Ming Ying, Ning Lyu

**Affiliations:** Department of Pathology, National Cancer Center/National Clinical Research Center for Cancer/Cancer Hospital, Chinese Academy of Medical Sciences and Peking Union Medical College, No.17 Panjiayuan Nanli, Chaoyang District, Beijing, 100021 People’s Republic of China

**Keywords:** Metastatic CRC, Gene mutation, Next-generation sequencing, *RAS*, *BRAF*, *PIK3CA*

## Abstract

**Purpose:**

To better understand the gene mutational status and heterogeneity between primary and metastatic CRC (mCRC) using a sensitive sequencing method.

**Methods:**

The mutational status of *EGFR, KRAS, NRAS, PIK3CA, ERBB2, BRAF, KIT*, and *PDGFRA* was analyzed in 65 patients, with 147 samples of primary and paired live or lung metastatic CRC, using next-generation sequencing (NGS), quantitative RT-PCR (qPCR), and Sanger sequencing.

**Results:**

Fifteen cases (15/22, 68.2%) of lung mCRC and thirteen cases (13/20, 65%) of liver mCRC harboured the same mutation profiles of *KRAS, NRAS*, or *BRAF* in the primary lesions. To all detected genes, 11 cases (11/22, 50%) of lung mCRC and 11 cases (11/20, 55%) of liver mCRC showed different mutational genes in the primary tumours. *KRAS* and *BRAF* mutations were more frequent in lung metastatic lesions (*p* = 0.004 and 0.003, respectively). The gene mutations in *KRAS, NRAS, BRAF*, and *PIK3CA* in the lung metastatic sites were more frequent than those in the liver metastatic sites (86.7 vs. 44%, respectively, *p* = 0.000). Some new mutations were not covered in the qPCR ranges but were detected by NGS.

**Conclusion:**

The study demonstrated that the discordance of gene mutational status between paired primary and metastatic tumours is rather high when detected by NGS. Evaluating the mutational status of both the primary and metastatic tumours should be considered in clinical mutation testing.

## Introduction

Colorectal cancer (CRC) is one of the most common malignancies, and it is the fifth leading cause of cancer-related deaths in China (Chen et al. 2016). Invasion and metastasis account for the high mortality rate of CRC. Targeted therapies have transformed the current strategies in tumour treatment. Patients with metastatic CRC (mCRC) have greatly benefited from antibody-mediated blockade of the epidermal growth factor receptor (EGFR), with antibodies such as cetuximab and panitumumab (Dietel et al. 2015).

The EGFR signalling pathway plays a pivotal role in the proliferation and survival of CRC (Efferth 2012). The targeted drug that blocks EGFR is a therapeutic option in the treatment of mCRC. However, mutations in the genes downstream of the EGFR signalling pathway result in a constitutive activation of the EGFR and thus might abolish the effects of EGFR antibodies (Sinicrope et al. 2016). *KRAS* and *NRAS* are some of these well-known genes. *BRAF* and *PIK3CA* mutations may also be negative predictive biomarkers for anti-EGFR therapies (Nakayama et al. 2017). Moreover, other cancer-related genes, such as *KIT* and *PDGFRA*, may also act as important predictive and prognostic markers in CRC (Li et al. 2016). Therefore, it is essential to accurately detect the mutational status of CRC. The selection of suitable tissue samples is critical for accurate mutation detection but is challenging for pathologists (Van Cutsem et al. 2016). Some studies reported that the concordance in *KRAS* mutational status between the primary and the metastatic tumours is high, and tissue from either the primary tumour or metastasis may be used for testing mutations according to the latest NCCN guidelines for the treatment of mCRC (Han et al. 2012). However, some studies find that spatial and temporal tumour heterogeneity, which exists between the primary and the metastatic tumours, is significant and thus accounts for targeted therapy failure (Goswami et al. 2015).

Next-generation sequencing has been widely used in mutation detection in recent years, as it provides high analytic sensitivity and a broad reportable range (Haley et al. 2015). NGS has become a robust tool for molecular diagnosis of CRC (Kothari et al. 2014; Malapelle et al. 2015). Moreover, NGS can provide a quantitative measurement of mutant allele frequencies and predict tumour heterogeneity. In this study, we compared the mutational status between primary CRC and paired liver or lung metastasis by NGS, qPCR, and Sanger sequencing assays. We hope that this study may help in better understanding the mutational status and heterogeneity in mCRC and selecting suitable samples and technology for the accurate mutation detection of CRC by pathologists.

## Materials and methods

### Patient selection

This study included patients from a consecutive pathology database with surgically resected liver or lung mCRC in the Department of Pathology, Cancer Hospital, Chinese Academy of Medical Sciences, and Peking Union Medical College from 2007 to 2015, whose primary CRC was also resected at this hospital. Both the primary and metastatic resected specimens could be obtained for testing. At the time of surgical resection of mCRC, the patients had only lung or liver metastasis, but other metastatic sites, such as peritoneal, brain, ovary, etc., were not excluded. Patients who underwent neoadjuvant therapy before resection of primary tumours were not chosen for the study. All patients were not treated with cetuximab or other targeted agents before or after surgical resections of the primary or metastatic CRC.

### Specimen selection

For each patient, every lesion of both primary and metastasis CRC was taken out, and the H&E slides were evaluated by two pathologists (SMZ and JMY). One of the representative paraffin blocks from each lesion was chosen for testing. A tumour content of more than 50% was usable for DNA extraction.

### Genomic DNA extraction and quantitation

The genomic DNA from no less than five consecutive, 5 µm-thick slides of FFPE samples was extracted using the QIAamp DNA FFPE Tissue Kit (Qiagen, Hilden, Germany) per the manufacturer’s instructions. The DNA concentration was determined by the genomic DNA quantitative detection kit (Beijing ACCB Biotech Ltd., Beijing, China) on a CFX96 RT-PCR instrument (Bio-Rad, California, USA), and it was normalized to approximately 20–50 ng/µl. The DNA samples were stored at − 20 °C before utilization.

### Mutation analysis by next-generation sequencing (NGS)

The NextDaySeq colon panel on the Ion TorrentTM System (Manufactured by Beijing ACCB Biotech, Beijing, China) was used to construct libraries according to the manufacturer’s instructions. Briefly, genomic regions of *EGFR* exons 18, 19, 20, and 21; *KRAS* exons 2 and 3; *NRAS* exons 2 and 3; *PIK3CA* exons 9 and 20; *ERBB2* exon 20; *BRAF* exons 11 and 15; *KIT* exons 9, 11, 13, 14, and 17; and *PDGFRA* exons 12, 14, and 18 were amplified with pooled primer pairs, followed by ligations of the adaptors and barcodes. The libraries were purified and quantified using a Qubit dsDNA HS Assay Kit on a Qubit 2.0 fluorometer (Thermo Fisher Scientific, Waltham, MA, USA); they were then diluted to a concentration of 3 ng/ml and pooled. The library pool was sequenced using the Ion Torrent PGM system. Briefly, the library pool was clonally amplified in an emulsion PCR reaction using Ion Sphere Particles on the OneTouch 2 instrument (Thermo Fisher Scientific). Template-positive ion sphere particles were enriched on the Ion OneTouch ES (Thermo Fisher Scientific). After enrichment, the sequencing primers and polymerase were added from the Ion PGM™ Sequencing Supplies 200 v2 Kit (Thermo Fisher Scientific). The libraries were loaded to generate sequencing data on the Ion 318 chips (Thermo Fisher Scientific) and sequenced on an Ion Torrent PGM (Thermo Fisher Scientific). A proprietary bioinformatics pipeline, the DanPA bioinformatics pipeline, was employed for variant calling and annotation. The pipeline was specifically designed for the NextDaySeq panels. The cutoff of mutation frequency for mutation calling was 1%, and that of mutation reads was 5.

### Mutation analysis by quantitative RT-PCR (qPCR)

Mutation profiles of *EGFR, KRAS, NRAS, BRAF*, and *PIK3CA* were detected using the corresponding Human Gene Mutations Detection Kit, Human KRAS Gene Mutations Detection Kit, Human NRAS Gene Mutations Detection Kit, Human BRAF Gene Mutations Detection Kit, and Human PIK3CA Gene Mutations Detection Kit (Real-time Fluorescent PCR, Beijing ACCB Biotech Ltd., Beijing, China), respectively. The tests cover 72 hotspot mutations including 45 in exons 18, 19, 20, and 21 of *EGFR*; 12 in exons 2 and 3 of *KRAS*; 9 in exons 2 and 3 of *NRAS*; 5 in exons 9 and 20 of *PIK3CA; BRAF* V600E. The quantitative PCR was performed on an Mx3000P PCR instrument (Agilent, CA, USA) with the following settings: 95 °C for 10 min; 40 cycles of 95 °C for 15 s and 60 °C for 1 min. The results were interpreted as per the manufacturer’s instructions.

### Mutation analysis by Sanger sequencing

The same genomic regions of eight genes covered in the NextDaySeq colon panel were amplified and sequenced bi-directionally with the ABI Prism Big Dye Terminator v 3.1 Cycle Sequencing Kit on an ABI 3130XL Genetic Analyzer (Thermo Fisher Scientific) according to the manufacturer’s instructions.

### Definition of the gene mutation type

The wild type was defined as having no gene mutations detected in the gene panel by any of the gene sequencing methods. The mutant type was defined as having any mutation detected in the gene panel by any of the gene sequencing methods. A single mutation was defined as having only one gene mutation in the gene panel detected by any of the gene sequencing methods. The combined mutation was defined as having more than one gene mutation in the gene panel detected by any of the gene sequencing methods.

### Statistics

Statistical analyses were performed with SPSS 25.0 software (SPSS, Chicago, IL). To evaluate the statistical significance of the differences observed between the groups, a Chi-square test was applied. To be specific, the Pearson’s chi-square test, the Fisher’s exact test, and continuity correction were used in different situations. All *P* values represent two-sided statistical tests and only those with less than 0.05 were considered significant.

## Results

### Basic characteristics of patients and primary CRC

Seventy-five patients with the primary and paired metastatic CRCs were chosen for the study. In ten of the cases, either the primary or the metastatic specimen failed the gene mutation test because of poor DNA quality. Finally, a total of 65 CRC patients, including 29 patients with lung metastasis and 36 patients with liver metastasis, were included in this study. They were divided into two groups according to the different metastasis locations. There were 42 males and 23 females; the median age was 58 years (30–78 years). No statistical difference was found between the two groups based on sex and age (*p* = 0.891). Some of the patients had more than one primary and metastatic CRC lesion. As a whole, 147 samples of primary CRC lesions and the paired metastases samples were analyzed.

There were 13 cases of primary CRC tumours that were located in the right half colon (caecum to transverse colon), 15 were located in the left half colon (descended to rectosigmoid colon), and 37 were located in the rectum. Fifty-six cases were well differentiated (G1/G2) and nine cases were poorly differentiated (G3/G4) according to the 2010 WHO classification of gastrointestinal tumours. Pathologic TNM classification was done according to the AJCC 7th. T3 took the majority between the two groups (48/65, 74%), followed by T4a (9/65, 14%). More than two-thirds of patients suffered from lymph node metastasis. Three cases had additional peritoneal metastasis besides liver metastasis. There was no significant difference between the two groups on the primary tumour characteristics, including location, differentiation, and T and N classifications. Twenty-nine and thirty-six cases had synchronous or metachronous distant metastasis, respectively. Synchronous metastasis was more common in the liver than it was in the lung metastasis group (*p* = 0.000). Therefore, the median time from primary tumour resections to metastasis resections was much longer in the lung metastasis group (23, 1–61 months) than that in the liver metastasis group (0, 0–35 months). The patient and primary tumour characteristics are summarized in Table 1.

**Table 1:** Baseline characteristics of patients and their primary CRC (*n* = 65)

	Cases with only lung metastasis (*N* = 29)	Cases with only liver metastasis (*N* = 36)	*P* value
Sex			
Male	19	23	0.891
Female	10	13	
Median age (range)	59 (30–78)	58 (36–75)	
Location of primary tumours			
Left half colon	7	8	0.528
Right half colon	4	9	
Rectum	18	19	
Differentiation			
G1/G2	27	29	0.122
G3/G4	2	7	
T stage			
2	4	2	0.168
3	19	29	
4a	6	3	
4b	0	2	
N stage			
0	9	6	0.566
1a	6	7	
1b	6	12	
2a	5	5	
2b	3	6	
M stage			
M0	26	15	0.000
M1a	3	18	
M1b	0	3	
TNM stages			
I	3	0	0.000
II	4	4	
III	19	11	
IV	3	21	
History of treatments			
None	23	19	0.027
Neoadjuvant therapy	1	0	
Adjuvant therapy	5	17	
Median time of duration of time between the resection of primary lesions and metastases (range m)	23 (1–61)	0 (0–35)	
Metastatic type			0.000
Synchronous	8	21	
Metachronous	21	15	

### Gene mutational status detected by qPCR, NGS, and Sanger sequencing

All the 147 samples were sequenced by both NGS and qPCR. Ninety-five samples were mutant type. The *KRAS, NRAS, BRAF, PIK3CA, EGFR, KIT*, and *PDGFRA* mutation detected by either NGS or qPCR was 54 (36.73%), 11 (7.48%), 6 (4.08%), 18 (12.24%), 3 (2.04%), 2 (1.36%), and 1 (0.68%), respectively. NGS detected more mutations than qPCR did (83 vs 76, respectively), especially for *PIK3CA*. NGS found 18 *PIK3CA* mutations, while qPCR only detected nine mutations. A total of 14 mutations were detected only by NGS, which are 1 *KRAS* L52P, 3 *NRAS* A91V, 1 *BRAF* G469A, 2*PIK3CA* E545A, 2 *PIK3CA* Q546K, 1 *EGFR* G735C, 1 *EGFR* G796S, 1 *KIT* W557*, 1 *KIT* P573L, and 1 *PDGFRA* M648fs (the details are shown in Table 2).

**Table 2:** Gene mutational status detected by qPCR and NGS

Gene	Total (*N* = 147) (%)	Single mutation (%)	Combined mutation (%)	Mutation by qPCR (%)	Mutation by NGS (%)	qPCR and NGS concordance (%)	New locus detected by NGS
*KRAS*	54 (36.73)	43 (29.25)	11 (7.48)	53 (36.05)	47 (31.97)	46 (85.19, *n* = 54)	1 *KRAS* L52P
*NRAS*	11 (7.48)	7 (4.76)	3 (2.04)	8 (5.44)	9 (6.12)	6 (54.54, *n* = 11)	3 *NRAS* A91V
*BRAF*	6 (4.08)	2 (1.36)	4 (2.72)	5 (3.40)	6 (4.08)	4 (66.67, *n* = 6)	1 *BRAF* G469A
*PIK3CA*	18 (12.24)	4 (2.72)	13 (8.84)	9 (6.12)	18 (12.24)	9 (50.00, *n* = 18)	2 2*PIK3CA* E545A, 2 *PIK3CA* Q546K
*EGFR*	3 (2.04)	2 (1.36)	1 (0.68)	1 (0.68)	3 (0.20)	2 (66.67, *n* = 3)	1 *EGFR* G735C, 1 *EGFR* G796S
*KIT*	2 (1.36)	2 (1.36)	0 (0)	0 (0)	2 (1.36)	0 (0, *n* = 2)	1 *KIT* W557*, 1 *KIT* P573L
*PDGFRA*	1 (0.68)	0 (0)	1 (0.68)	0 (0)	1 (0.68)	0 (0, *n* = 1)	1 *PDGFRA* M648fs
Total	95 (64.63)	60 (40.82)	33 (22.45)	76 (80.00)	83 (87.37)	67 (70.53)	14

Sanger sequencing was performed in the first 16 tested cases, with 32 samples. Only 20 samples, including 9 wild type and 11 mutant type, showed the same results with NGS or qPCR method. Twelve samples were not consistent with NGS or qPCR results, which were nine samples mutant type in NGS or qPCR, but were wild type in Sanger sequencing. The other three cases were no results by Sanger sequencing. In addition, no mutations that were found by Sanger sequencing were not detected by NGS or qPCR. The results showed that the low sensitivity of Sanger sequencing compared with the other two methods.

### Gene mutations in the primary and the metastatic CRC

Twenty-nine cases, with fifty-nine paired primary and lung metastatic CRC tissues, were analyzed. One case (No. 20) had two lung metastatic loci. Both the primary and the paired lung metastatic CRC were wild type in 7 patients (7/29, 24.1%). Gene mutations were found in the left 22 cases, 18 (18/29, 62.1%) in the primary sites, and 20 (20/30, 66.7%) in the metastatic sites. A total of 46 gene mutations were found, 20 (20/46, 43.5%) in the primary sites, and 26 (26/46, 56.5%) in the metastatic sites. In the primary sites, the gene mutations included 14 in *KRAS*, 2 in *NRAS*, 1 in *PIK3CA*, 2 in *BRAF*-V600E, 1 in *KIT* W557*, 1 in *PDGFRA* M648fs. The gene mutations in the metastatic sites were 17 in *KRAS*, 1 in *NRAS*, 3 in *PIK3CA*, 4 in *BRAF*, and 1 in *EGFR* (the details are shown in Table 3). The *KRAS* mutations were most frequent both in the primary and the metastatic CRCs (13/20, 65% and 17/26, 65.4%, respectively). Some mutations were detected by NGS and not included in the qPCR genomic regions, which were *KIT* W557*, *PDGFRA* M648fs, *KRAS* L52P, and *BRAF* G469A.

**Table 3:** Gene mutations in the primary and the metastatic CRC

Mutant genes	Primary site of CRC with lung metastasis (*n* = 29)	Primary site of CRC with liver metastasis (*n* = 38)	*P* value	Metastatic site of CRC with lung metastasis (*n* = 30)	Metastatic site of CRC with liver metastasis (*n* = 50)	*P* value
*KRAS*	13	13	0.377	17	11	0.002
* KRAS* G12D	5	7	0.901	5	6	0.557
* KRAS* G13D	6	2	0.121	6	2	0.054
* KRAS* G12V	0	3		2	1	
* KRAS* G12R	1	0		1	0	
* KRAS* G12A	1	0		1	0	
* KRAS* G12C	0	0		1	0	
* KRAS* Q61H	0	1		0	2	
* KRAS* L52P	0	0		1	0	
*NRAS*	2	5	0.406	1	3	0.596
* NRAS* Q61K	1	0		0	0	
* NRAS* G12D	1	2		1	2	
* NRAS* Q61R	0	1		0	0	
* NRAS* A91V	0	2		0	1	
*BRAF*	2	0	0.184	4	0	0.034
* BRAF* V600E	2	0		3	0	
* BRAF* G469A	0	0		1	0	
*PIK3CA*	1	8	0.083	3	6	1.000
* PIK3CA* E545A	1	1		0	0	
* PIK3CA* E545K	0	3		0	1	
* PIK3CA* Q546K	0	1		0	1	
* PIK3CA* E542K	0	1		2	1	
* PIK3CA* H1047R	0	2		1	3	
*KIT* W557*	1	0		0	0	
* KIT* P573L	0	0		0	1	
* PDGFRA* M648fs	1	0		0	0	
* EGFR* G735C	0	1		0	0	
* EGFR* L858R	0	0		1	0	
* EGFR* G796S	0	0		0	1	
Total	20	27	0.853	26	22	0.000

Thirty-six cases, with eighty-eight paired primary and liver metastatic CRC, were analyzed. Two cases had two primary sites; and three, two, and one cases had two, three, and eight metastatic sites, respectively. Both the primary and paired liver metastatic CRC were wild type in 16 patients (16/36, 44.4%). Gene mutations were found in 20 cases, 18 (18/20, 90%) from the primary site, and 17 (17/20, 85%) from the metastatic sites. A total of 49 gene mutations were found: 27 (27/49, 55.1%) from the primary sites and 22 (22/49, 44.9%) from the metastatic sites. There were more gene mutations in the primary sites than those in the metastatic sites. In the primary sites, gene mutations included 13 in *KRAS*, 5 in *NRAS*, 8 in *PIK3CA*, and 1 in *EGFR*. The gene mutations in the metastatic sites were 11 in *KRAS*, 3 in *NRAS*, 6 in *PIK3CA*, 1 in *KIT*, and 1 in *EGFR*. No *BRAF* mutation was found in either the primary or the liver metastatic CRC (the details are shown in Table 3). Some mutations were detected by NGS and were not included in the qPCR genomic regions, which were *KIT* P573L, *EGFR* G735C, and *EGFR* G796S.

When comparing the mutational gene status between the primary site of CRC with lung and liver metastasis (the details are shown in Table 3), we found that there was no significant difference in *KRAS, NRAS, BRAF*, and *PIK3CA*. Regarding the metastatic site, gene mutations in the lung metastatic sites were significantly much more prevalent than those in the liver metastatic sites (*p* = 0.000), with a total 26 gene mutations (26/30, 86.7%) and 22 gene mutations (22/50, 44%), respectively. We also found 17 and 11 *KRAS* gene mutation in the lung metastasis and liver metastasis, respectively (56.7 vs. 22%, *p* = 0.002).

### The mutational consistency between paired primary and lung metastatic CRC

Fifteen cases (15/22, 68.2%) harboured the same mutation profiles between the primary and the lung metastatic lesions in *KRAS, NRAS*, and *BRAF*, which were 4 *KRAS* G12D, 6 *KRAS* G13D, 1 *KRAS* G12R, 1 *KRAS* G12A, 1 *NRA*S G12D, and 2 *BRAF* V600E. All were CRCs with metachronous metastasis. Eleven cases (11/22, 50%) harboured the exactly the same mutation between the primary and the lung metastatic lesions. The other 11 cases (11/22, 50%) showed somewhat different mutational genes (the details are shown in Table 4). Five of these cases showed gene mutation discrepancies beside *KRAS, NRAS*, and *BRAF*, which were *EGFR* L858R, *PIK3CA* H1047R, *PIK3CA* E542K, *PDGFRA* M648fs, and *KIT* W557*. In the other six cases, the gene mutation profiles were different in *KRAS, NRAS*, or *BRAF*, and five of them were metachronous metastasis. Only five cases underwent adjuvant chemotherapy or radiochemotherapy after primary tumour was resected, the gene mutation inconsistent cases were not statistical different between adjuvant and non-adjuvant therapy groups.

**Table 4:** Mutational cases in paired primary and lung metastatic CRC (*n* = 22)

Mutational consistency	No.	Case no.	Mutational type		Synchronous (S)/metachronous (M) metastasis	Adjuvant(A)/non-adjuvant (N) therapy
			Primary site	Metastatic site		
Mutation consistent cases (*n* = 11)	1	Lung01	*KRAS* G13D	*KRAS* G13D	M	N
	2	Lung04	*KRAS* G12D	*KRAS* G12D	M	N
	3	Lung07	*KRAS* G12D	*KRAS* G12D	M	A
	4	Lung09	*KRAS* G13D	*KRAS* G13D	M	N
	5	Lung12	*KRAS* G12D	*KRAS* G12D	M	A
	6	Lung13	*KRAS* G13D	*KRAS* G13D	M	N
	7	Lung21	*KRAS* G12R	*KRAS* G12R	M	N
	8	Lung23	*KRAS* G12A	*KRAS* G12A	M	N
	9	Lung24	*KRAS* G13D	*KRAS* G13D	M	N
	10	Lung25	*KRAS* G12D	*KRAS* G12D	M	N
	11	Lung26	*NRAS* G12D	*NRAS* G12D	M	N
Mutation inconsistent cases beside *KRAS, NRAS* or *BRAF* (*n* = 5)	12	Lung06	*KRAS* G13D	*KRAS* G13D, *EGFR* L858R	M	A
	13	Lung10	*KRAS* G13D	*KRAS* G13D, *PIK3CA* H1047R	M	N
	14	Lung20	*BRAF* V600E	M1^a^: *BRAF* V600E, *PIK3CA* E542K M2^b^: *BRAF* V600E, *PIK3CA* E542K	M	N
	15	Lung15	*BRAF* V600E *PDGFRA* M648fs	*BRAF* V600E	M	N
	16	Lung03	*KIT* W557*	Wild type	M	N
Mutation inconsistent cases in *KRAS, NRAS and BRAF* (*n* = 6)	17	Lung18	Wild type	*KRAS* G12D	M	N
	18	Lung19	Wild type	*KRAS* G12D	M	N
	19	Lung27	Wild type	*KRAS* G12C	S	N
	20	Lung28	Wild type	*KRAS* L52P, *BRAF* G469A	M	N
	21	Lung17	*KRAS* G12D *PIK3CA* E545A	Wild type	M	A
	22	Lung14	*NRAS* Q61K	*KRAS* G12D	M	N

^a^Lung metastatic site 1

^b^Lung metastatic site 2

### The mutational consistency between paired primary and liver metastatic CRC

Thirteen cases (13/20, 65%) harboured the same mutation profiles between the primary and the liver metastatic lesions in *KRAS, NRAS*, and *BRAF*, which were *KRAS* G12D, 1 *KRAS* G13D, 1 *KRAS* G12V, 1 *KRAS* Q61H, 1 *NRAS* G12D, 1 *NRAS* A91V, 1 *PIK3CA* E542K, 1 *KRAS* G13D and *PIK3CA* Q546K, and 1 *PIK3CA* H1047R and *NRAS* G12D. Nine cases (9/20, 45%) harboured the exactly the same mutation between the primary and the liver metastatic lesions. The other 11 cases (11/20, 55%) showed somewhat different mutational genes (the details are shown in Table 5). Six of these cases showed gene mutation discrepancies beside *KRAS, NRAS*, and *BRAF*, which were *PIK3CA* E545K, *PIK3CA* H1047R, *EGFR* G796S, and *KIT* P573L. In the other five cases, the gene mutation profiles were different in *KRAS, NRAS*, or *BRAF*. Most of these mutation inconsistent cases were with synchronous metastasis (9/11, 81.8%), there were 17 cases underwent adjuvant therapy. The mutation inconsistent cases were more in non-adjuvant therapy group than in adjuvant therapy group (9/19 vs 2/17, *P* = 0.021).

**Table 5:** Mutational cases in paired primary and liver metastatic CRC (*n* = 20)

Mutational consistency	No.	Case no.	Mutation types		Synchronous (S) or metachronous (M) metastasis	Adjuvant(A) or non-adjuvant (N) therapy
			Primary site	Metastatic site		
Mutation consistent cases (*n* = 9)	1	Liver 02	*NRAS* G12D *PIK3CA* H1047R	*NRAS* G12D *PIK3CA* H1047R	S	A
	2	Liver 03	*KRAS* G12D	*KRAS* G12D	S	N
	3	Liver 10	*NRAS* G12D	*NRAS* G12D	M	A
	4	Liver 12	*KRAS* Q61H	*KRAS* Q61H	S	N
	5	Liver 19	P1^a^: *KRAS* G12D P2^b^: *KRAS* G12D	*KRAS* G12D	M	A
	6	Liver 21	*PIK3CA* E542K	*PIK3CA* E542K	M	A
	7	Liver 22	*KRAS* G12V	*KRAS* G12V	M	A
	8	Liver 25	*KRAS* G12D	*KRAS* G12D	M	N
	9	Liver 27	*KRAS* G13D *PIK3CA* Q546K	*KRAS* G13D *PIK3CA* Q546K	M	A
Mutation inconsistent cases beside *KRAS, NRAS* or *BRAF* (*n* = 6)	10	Liver 01	*KRAS* G12D *PIK3CA* E545K	*KRAS* G12D	M	N
	11	Liver 14	*KRAS* G12D *PIK3CA* H1047R *PIK3CA* E545K	*KRAS* G12D *PIK3CA* H1047R	S	N
	12	Liver 23	*KRAS* G13D	*KRAS* G13D *PIK3CA* H1047R	M	N
	13	Liver 04	Wild type	M1^c^ *KIT* P573L M2^d^ wild type	S	A
	14	Liver 16	Wild type	*EGFR* G796S	S	N
	15	Liver 18	*PIK3CA* E545K	Wild type	S	A
Mutation inconsistent cases in *KRAS, NRAS* or *BRAF* (*n* = 5)	16	Liver 34	*KRAS* G12D *PIK3CA* E545K	*PIK3CA* E545K	S	N
	17	Liver 07	*KRAS* G12V	Wild type	S	N
	18	Liver 09	*KRAS* G12V	Wild type	S	N
	19	Liver 13	*EGFR* G735C	*KRAS* G12D	S	N
	20	Liver 28	P1^a^ *NRAS* A91V P2^b^ *NRAS* A91V *NRAS* Q61R	*NRAS* A91V	S	N

^a^P1: primary site 1

^b^P2: primary site 2

^c^M1: liver metastatic site 1

^d^M2: liver metastatic site 2

### Gene mutation discrepancies among the separate primary or metastatic site(s) in one case

For those cases with more than one primary or metastatic site, each site was sequenced separately. We did observed that gene mutational status discrepancies were exist in CRC with liver metastasis. The details were summarized in Table 6. Case Liver 04 was a rectal cancer patient who suffered multiple simultaneous liver metastases in the sixth and seventh hepatic segment. The patient had a *KIT* P573L mutation only in the seventh hepatic segment lesion, while both the primary and the sixth hepatic segment metastatic lesions were wild type. Case Liver 28 was a patient with two primary lesions in the rectum and one simultaneous liver metastatic lesion in the seventh hepatic segment. The *NRAS* A91V mutation could be detected in all three lesions in this case, while the *NRAS* Q61R mutation was only detected in one of the primary lesions.

**Table 6:** Gene mutational status among the multiple primary or metastatic site(s) in one case

Case no.	Location	Gene mutational status	Synchronous(S)/metachronous(M) metastasis	Gene mutation consistency among each site (Yes; Y or No: N)
Liver^a^ 04	P^b^	Wild type	S	N
	M^c^1^d^	*KIT* P573L		
	M2	Wild type		
Liver 28	P1	*NRAS* A91V	S	N
	P2	*NRAS* A91V,*NRAS* Q61R		
	M	*NRAS* A91V		
Lung^e^ 20	P	*BRAF* V600E	M	Y
	M1	*BRAF* V600E, *PIK3CA* E542K		
	M2	*BRAF* V600E, *PIK3CA* E542K		
Liver 12	P	*KRAS* Q61H	S	Y
	M1, M2	*KRAS* Q61H		
Liver 19	M	KRAS G12D	M	Y
	P1, P2	KRAS G12D		
Liver 15	M1-M3	Wild type	M	Y
	P	Wild type		
Liver 17	M1-M8	Wild type	M	Y
	P	Wild type		
Liver 31	M1-M3	Wild type	S	Y
	P	Wild type		
Liver 33	M1, M2	Wild type	S	Y
	P	Wild type		

^a^CRC with liver metastasis

^b^Primary site

^c^Metastatic site

^d^Numbers of each primary or metastatic site(s)

^e^CRC with lung metastasis

## Discussion

*KRAS* and *NRAS* mutations are critical negative predictors of anti-EGFR therapy. In addition to *KRAS* and *NRAS* mutations, *BRAF* and *PIK3CA* mutations may also be potential biomarkers of resistance to anti-EGFR targeted treatments (Hsu et al. 2016; Karthaus et al. 2016). Some studies find that high concordance can be observed between the primary and metastatic tumours, suggesting that the detection of one lesion is sufficient to predict the response to EGFR-targeted therapy (Artale et al. 2008; Knijn et al. 2011; Santini et al. 2008). Therefore, the latest NCCN guideline strongly recommends genotyping of tumour tissue (either primary tumour or metastasis) in all patients with metastatic colorectal cancer for *RAS* and *BRAF*. However, most of these reports perform the mutation test using Sanger sequencing or ARMS-PCR, which may be limited by low sensitivity or detection of common mutations (Artale et al. 2008; Knijn et al. 2011; Santini et al. 2008). Compared with these traditional technologies, NGS can provide higher sensitivity and border detection ranges. Therefore, using NGS may better uncover the genetic heterogeneity between primary and metastatic tumours.

In this study, we detected the mutation profile between paired primary and liver or lung metastatic tumours using multiple sequencing methods. In general, NGS detected more mutations than qPCR did. We found that the mutation profile discordant rate was high between primary and metastatic CRC, which may influence the treatment decision on anti-EGFR target therapy. There were 11 cases (11/22, 50%) which showed different mutational genes between the primary and the lung metastatic tumours. The gene mutation profiles were different in *RAS* or *BRAF* in six of these cases. In four cases, tumours were wild type in primary sites, but were *RAS* mutant type in lung metastatic sites; in another 1 case, gene mutation changed from *KRAS* G12D and *PIK3CA* E545A in primary site to wild type in lung metastatic site. There were also 11 cases (11/20, 55%) which showed different mutational genes between the paired primary and liver metastatic CRCs. The gene mutation profiles were different in *RAS* or *BRAF* in five of these cases. Three cases were *RAS* mutant type in primary sites, but were *RAS* wild type in metastatic sites; on the contrary, another 1 case was *RAS* wild type in primary site, but was *RAS* mutant type in metastatic site. We also found that mutation inconsistent cases were much more in CRC with synchronous liver metastasis. These data strongly support the notion that testing the mutational status of metastatic tumours is necessary rather than only testing the mutational status of primary tumours to further guide targeted therapy selection, both in synchronous and metachronous metastatic cases.

Neoadjuvant or adjuvant radiochemotherapy may have potential effect on gene mutation status. Some studies tried to determine if there are any changes in *KRAS* mutation status before and after preoperative chemoradiotherapy (CRT) in primary CRC. The results were controversial. In the study by Boissiere-Michot et al., *KRAS* mutation was identified in 9/31 (29%) surgical specimens which major tumour regression is achieved in locally advanced rectal adenocarcinomas after radiochemotherapy, less than in the paired pretreatment biopsies (12/31, 39%) (Boissiere-Michot et al. 2012). Jo et al. did not obtain evidence that CRT results in changes of the *KRAS* mutation pattern and no intratumoural heterogeneity in the *KRAS* mutational status could be proven before and after neoadjuvant CRT. The discrepancy observed in these studies may be caused by the amount of viable tumour cells after CRT and the sensitivity of the analytical method (Jo et al. 2016). The gene mutation discrepancy between primary and metastatic tumour with or without adjuvant therapy is unknown. In the present study, there were 17 cases which underwent adjuvant chemotherapy in liver metastatic CRC group. We observed that the mutation inconsistent cases were more in non-adjuvant therapy group than in adjuvant therapy group (9/19 vs 2/17, *P* = 0.021). This data may show potential therapy-related changes, but it needs to be proven by further larger and well-designed studies.

The presence of tumour heterogeneity has been found in many cancers and is a possible explanation for therapy resistance (Xie et al. 2014). Several studies have demonstrated the genetic, epigenetic, and transcriptomic heterogeneity in CRC (Jones et al. 2017; Punt et al. 2017). In our study, we tested the mutational status between 65 paired primary and metastatic tumours, including 36 paired primary-liver metastatic tumours and 29 paired primary-lung metastatic tumours. We found that the discordant rate of mutational status between the paired primary and the metastatic tumours was as high as 55%. In addition, more gene mutations were detected in the lung metastatic tumours than those in the primary tumours. The possible explanation is that most lung metastatic tumours are metachronous metastasis. Discordant mutations may be acquired in the lung metastases because of clonal evolution following chemotherapy compared with that in the primary tumours (Findlay et al. 2016).

In addition to predicting the response to EGFR-targeted therapy, the *KRAS, NRAS, BRAF*, and *PIK3CA* mutations may also act as significant prognostic factors in CRC (Foltran et al. 2015; Vogelaar et al. 2015). Here, we found that the frequency of *KRAS* and *BRAF* mutations was higher in the lung metastatic tumours than that in the liver metastatic tumours. These data indicate that determining the mutation profiling of CRC may have a potential implication for CRC development and progression.

Moreover, some rare gene mutations were observed in our studies, such as the *KIT* and *EGFR* mutations. These rare mutations may also act as a potential target for the treatment of CRC, and application of NGS can comprehensively determine the mutational status of CRC (Chen et al. 2015; Cho et al. 2014). However, more studies are necessary to further explore the relationship between these rare mutations and target therapies.

This study has some limitations. First, it is a retrospective study of a single centre. Second, some samples were excluded for poor DNA quality and the number of samples was relatively small. Third, only the resection samples were in the study, which may show case selection bias, because those were all resectable or potentially resectable mCRC, which were in a relatively early stage.

In conclusion, our study demonstrates that the discordance of the gene mutational status between paired primary and metastatic CRC is rather high when detected by NGS. Therefore, evaluating the mutational status of both the primary and the metastatic tumours should be considered in clinical mutation testing to better guide targeted therapies of mCRC.
